# Comparison of pain and swelling after removal of oral leukoplakia with CO2 laser and cold knife: A randomized clinical trial

**DOI:** 10.4317/medoral.17960

**Published:** 2012-12-10

**Authors:** Pía López-Jornet, Fabio Camacho-Alonso

**Affiliations:** 1 Full Professor of Oral Medicine. University of Murcia (Spain)

## Abstract

Objective: The aim of this study was to compare conventional surgery with carbon dioxide (CO2) laser in patients with oral leukoplakia, and to evaluate the postoperative pain and swelling. Study design: A total of 48 patients (27 males and 21 females) with a mean age of 53.7 ± 11.7 years and diagnosed with oral leukoplakia were randomly assigned to receive treatment either with conventional surgery using a cold knife or with a CO2 laser technique. A visual analog scale (VAS) was used to score pain and swelling at different postoperative time points. Results: Pain and swelling reported by the patients was greater with the conventional cold knife than with the CO2 laser, statistically significant differences for pain and swelling were observed between the two techniques during the first three days after surgery. Followed by a gradual decrease over one week. In neither group was granuloma formation observed, and none of the patients showed malignant transformation during the period of follow-up. Conclusions: The CO2 laser causes only minimal pain and swelling, thus suggesting that it may be an alternative method to conventional surgery in treating patients with oral leukoplakia.

** Key words:**Oral leukoplakia, treatment, laser surgery, cold knife, pain, swelling.

## Introduction

Oral leukoplakia is a predominantly white lesion of the oral mucosa that cannot be characterized as any other definable lesion; some oral leukoplakias will transform into cancer (1-6). The estimated worldwide prevalence of oral leukoplakia is approximately 2% (3). There are some geographical differences with regard to the gender distribution of the disease (2), and leukoplakia is moreover much more common among smokers than among non-smokers. Alcohol is thought to be an independent risk factor, but definitive data are still lacking. Actually, there are conflicting results of studies related to the possible role of human papillomavirus (HPV) infection (1,5). Histologically, leukoplakia is an intraepithelial lesion consisting of epithelial hyperplasia, with or without hyperkeratosis and minimal inflammation, and with or without varying degrees of dysplasia (2,6).

As regards the treatment of oral leukoplakia, intervention first should focus on the associated risk factors: with the suppression of smoking, alcohol abuse, and local irritating factors and overinfections. The second intervention, when the associated risk factors have been eliminated, it must be the oral biopsy. A number of treatment regimens have been proposed for oral leukoplakia without dysplasia, including surgical techniques such as scalpel excision, electrocoagulation, cryotherapy and laser CO2 therapy and also medical treatments such as vitamin A and retinoids, antioxidants and topical bleomycin (1,7-17). Leukoplakia is considered to be the most common potentially malignant disorder of the oral mucosa, the reported malignant transformation rates vary from 1.2-9% and the recurrence rates for oral leukoplakia vary from 7.7-38.1% (18-25).

The CO2 laser is currently accepted as the technique of choice in the management of oral leukoplakia and some malignant oral lesions (26), compared with the cold scalpel. In this context, the CO2 laser affords a haemostatic effect (in coagulation or haemostatic mode) particularly useful in highly vascularized areas, this result in a clearer surgical field and therefore a reduction in operating time. This laser can also be used for the treatment of superficial oral mucosa lesions (in section or cut mode).

Some authors have examined postoperative pain in the surgical treatment of soft tissue lesions (11,20), though there are few comparative studies on the different surgical techniques in relation to postoperative pain and swelling in oral leukoplakia patients.

The aim of the present study was to compare conventional cold knife surgery with the CO2 laser in patients with oral leukoplakia, and to evaluate pain and swelling during the first postoperative week.

## Material and Methods

-Study subjects

A total of 48 patients with oral leukoplakia who went to the Department of Oral Medicine of the University Clinic of Dentistry of the University of Murcia (Spain) between January of 2005 and December of 2010 were enrolled into the study, and the study was approved by the Ethics Committee of the University of Murcia. All patients had an incisional biopsy performed under local anesthesia prior to surgical treatment. The clinical diagnosis of the lesions was based on the criteria proposed by Axell et al. (5), adopted by the World Health Organization (WHO), and the histopathological diagnosis of epithelial dysplasia was established according to WHO definitions (3).

Informed consent was obtained from all patients before inclusion in the study. The patients received a complete physical head and neck examination. The possible etiological factors smoking and alcohol were identified, and advice on risk factor elimination was given before surgical treatment. The lesions were classified according to location and clinical appearance. All surgeries were performed by the same surgeon and carried out under local anesthesia with 4% articaine and 1:100,000 adrenalin (Inibsa; Lliça de Vall, Barcelona, Spain).

The patients were randomized (with an informatics system) by receiving stamped envelopes containing the numbers 1 or 2, indicating which group they would be placed in. Group 1 (17 males and 11 females) was treated by standard cold knife surgery. Group 2 (10 males and 10 females) was treated by CO2 laser (Lasersat 20W, Satelec®, Pierre Rolland, SATELEC®, S.A., Barcelona, Spain). Appropriate safety regulations for the use of lasers were followed by the operating personnel and patients (protective glasses and limited access to the surgical area).

**Figure 1:**
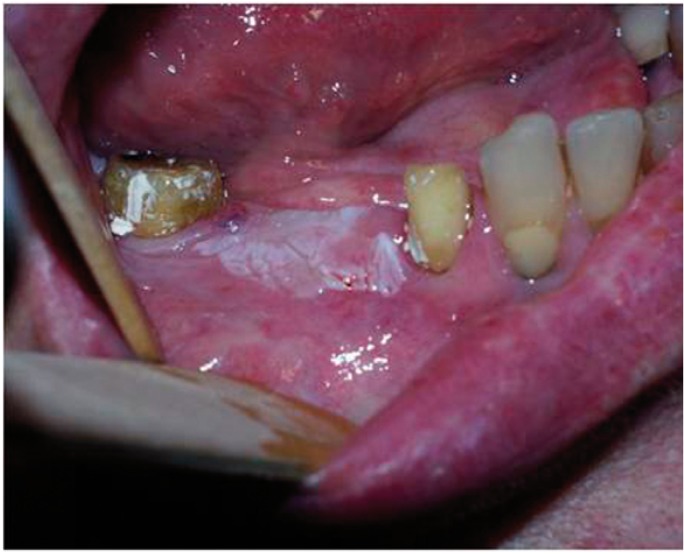
Oral leucoplakia of the gums (before the surgical treatment).

**Figure 2:**
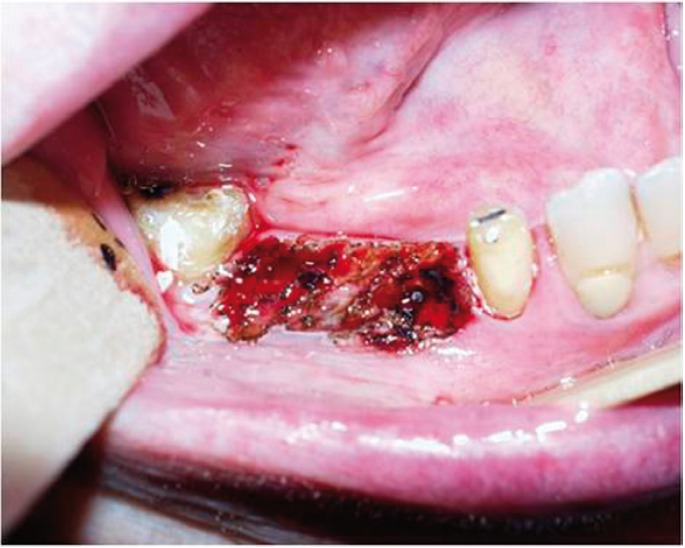
Oral leukoplakia of the gums (immediately after the surgical treatment with CO2 laser).

In group 1, the lesions were excised with a cold knife including 3 mm of clinically normal mucosa at the periphery. In group 2, the lesions were excised with CO2 laser in continuous mode, defocalized, with 15 mm of distance focus-lesion and a power setting of 5-15 W (5-15 seconds) (including, 3 mm of clinically normal mucosa at the periphery) (Figs. 1,2).

In group 1, the wounds were closed with 3/0 sterile suture (Laboratories Normon®, Madrid, Spain); but in group 2 the wounds regeneration were by second intention healing.

A VAS of 100 mm in length was used to evaluate the intensity of pain and swelling. This scale converted the visual analogical evaluation made by the patient into a numerical value: from 0 (corresponding to 0 mm on the VAS and showed no pain and no swelling) to 10 (100 mm on the VAS and showed unbearable pain and maximal swelling). Each patient was told how to use the VAS and was asked to mark the intensity of pain and swelling 12 and 24 hours after surgery and then on each day for the follow-ing 6 days. After the surgical intervention, 600 mg of Ibuprofen was prescribed every 8 hours for the first four postoperative days. Finally, the patients were revised at 7 days after treatment.

-Statistical analysis

Data were analyzed using the SPSS version 12.0 statistical package (SPSS® Inc., Chicago, IL, USA). A descriptive study was made of each variable. The Kolmogorov-Smirnov normality test and Levene variance homogeneity test were applied, and the data showing a skewed distribution were analyzed using a nonparametric ranking test. The associations between the different qualitative variables were studied using Pearson’s chi-squared test. We used the Kruskal-Wallis test (for more than two samples), the Mann-Whitney U-test (for two independent samples) and the Wilcoxon test (for two related samples), for quantitative variables. A probability p£0.05 was accepted as significant.

## Results

The mean sample age was 53.7 ± 11.7 years (range 28-74). As regards the oral leukoplakia risk factors, 39 of the 48 patients included in the study (81.3%) were cigarette smokers while 9 (18.8%) were non-smokers. Alcohol consumption was generally mild: social alcohol consumption (1 or 2 units per day) was recorded for 17 patients (35.4%), while 31 reported no alcohol intake (64.6%).

With respect to the clinical presentation of the lesions, 46 were homogeneous leukoplakias and 2 were no-homogeneous leukoplakias.

The lesions were distributed as follows: 10 in cheek mucosa (20.8%), 24 in gums (50%), 8 in palate (16.8%), 3 in tongue (6.2%) and 3 in floor of the mouth (6.2%).

The two study groups (cold knife and CO2 laser) were homogeneous in terms of patient age (p=0.592), gender (p=0.461), location (p=0.257), extent of the lesions (p=0.072) and the presence of dysplasia (p=0.471) ( Table 1).

**Table 1:**
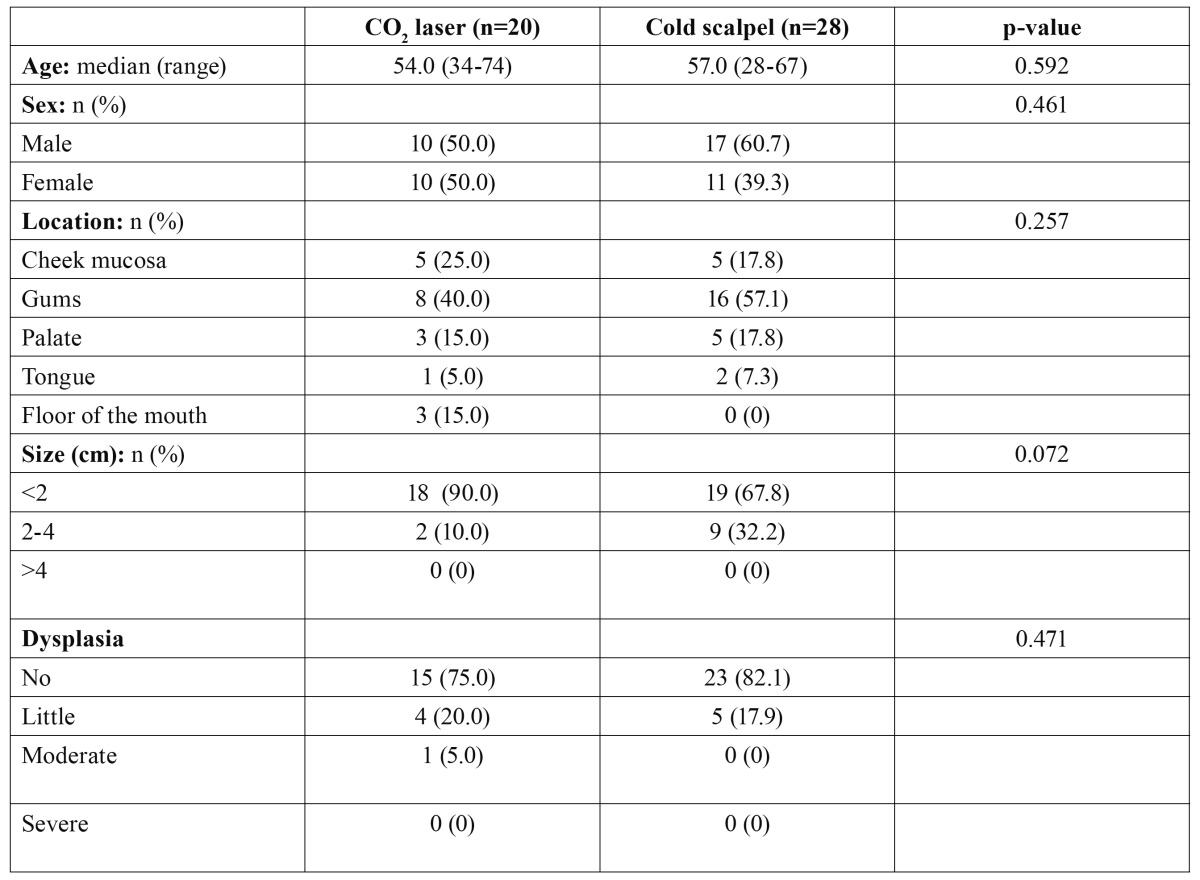
Homogeneity of the study groups in terms of the demographic characteristics, lesion location, size, and histological characteristics (Mann-Whitney U-test and Pearson χ2 test).

**Figure 3:**
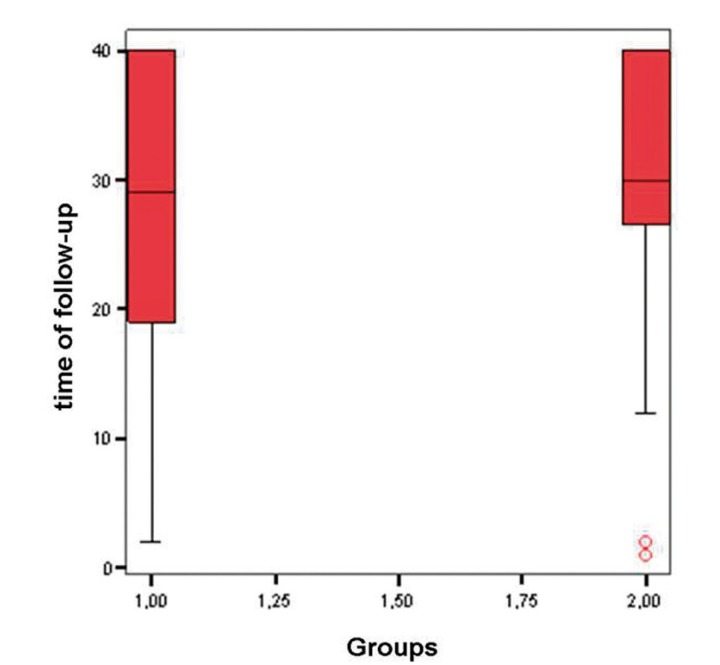
Box-plot with the time of follow-up of both study groups (group 1 = cold knife and group 2 = CO2 laser).

The mean duration of follow-up was 27.90 ± 12.05 months (range 1-40) (Fig. 3). In neither group was granuloma formation ob-served, and none of the patients showed malignant transformation during the period of follow-up.

Pain and swelling reported by the patients was greater with the cold knife than with the CO2 laser, with statistically significant differences between the two techniques during the first three days after surgery (p-value for related samples at 12h, 1, 2 and 3 days ≤ 0.05). Followed by a gradual decrease over one week in both groups ( Table 2).

**Table 2:**
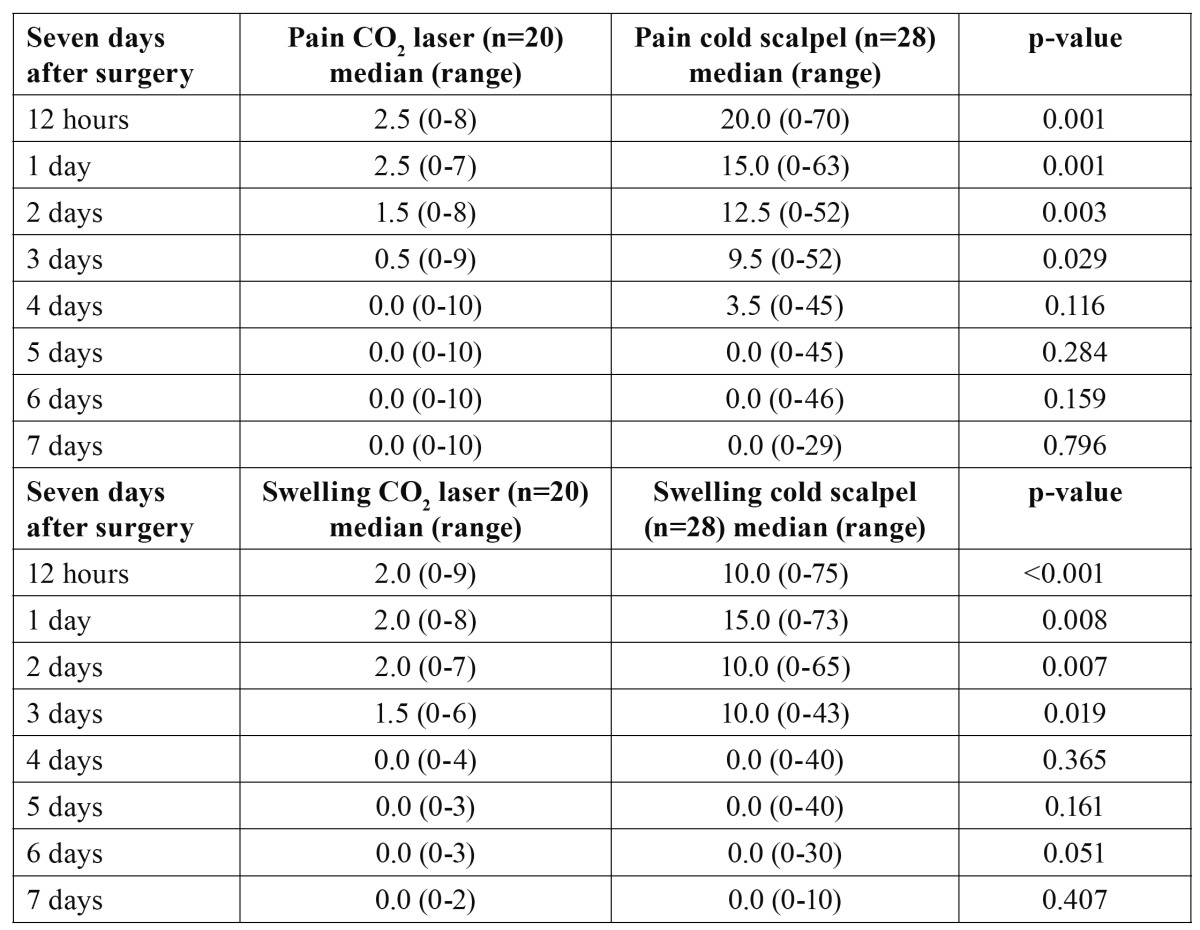
Incidence and severity of pain and swelling during the 7 days after oral leukoplakia removal (Mann-Whitney U-test).

In the CO2 laser group, on comparing the median (range) pain scores (recorded at peak pain intensity: between 12-24 hours after surgery) and swelling scores (recorded at peak swelling intensity: between 12-48 hours after surgery), statistically significant differences were observed for pain (p=0.021) and swelling (p=0.019) with respect to lesion size ( Table 3).

**Table 3:**
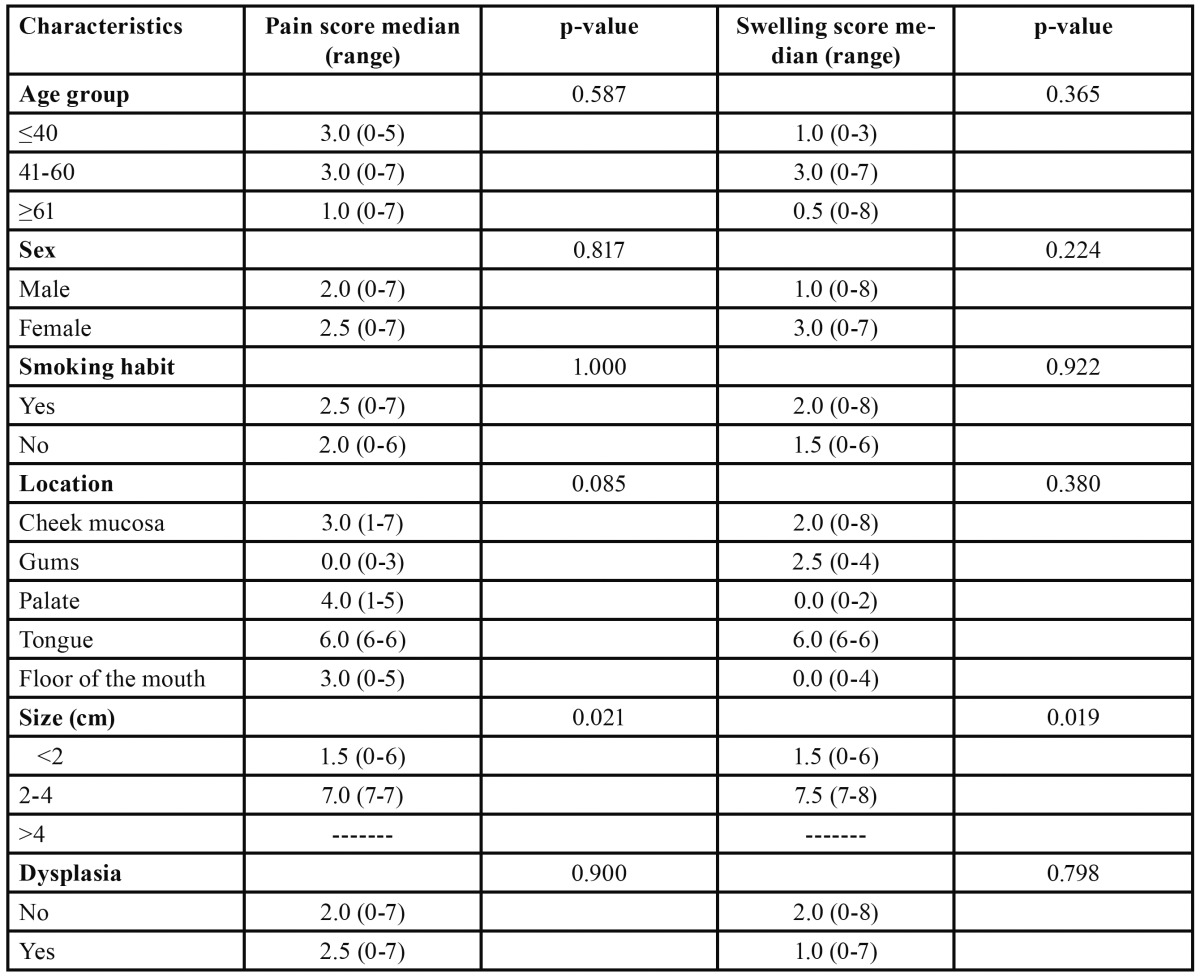
Comparison of median (range) pain scores (recorded at peak pain intensity: between 12-24 hours after surgery) and swelling scores (recorded at peak swelling intensity: between 12-48 hours after surgery) in the CO2 laser group (Kruskal-Wallis and Mann-Whitney U-test).

In turn, in the cold knife group, on comparing the median pain scores (recorded at peak pain intensity: 12 hours after surgery) and swelling scores (recorded at peak swelling intensity: 24 hours after surgery) no statistically significant differences were observed for the variables studied ( Table 4).

**Table 4:**
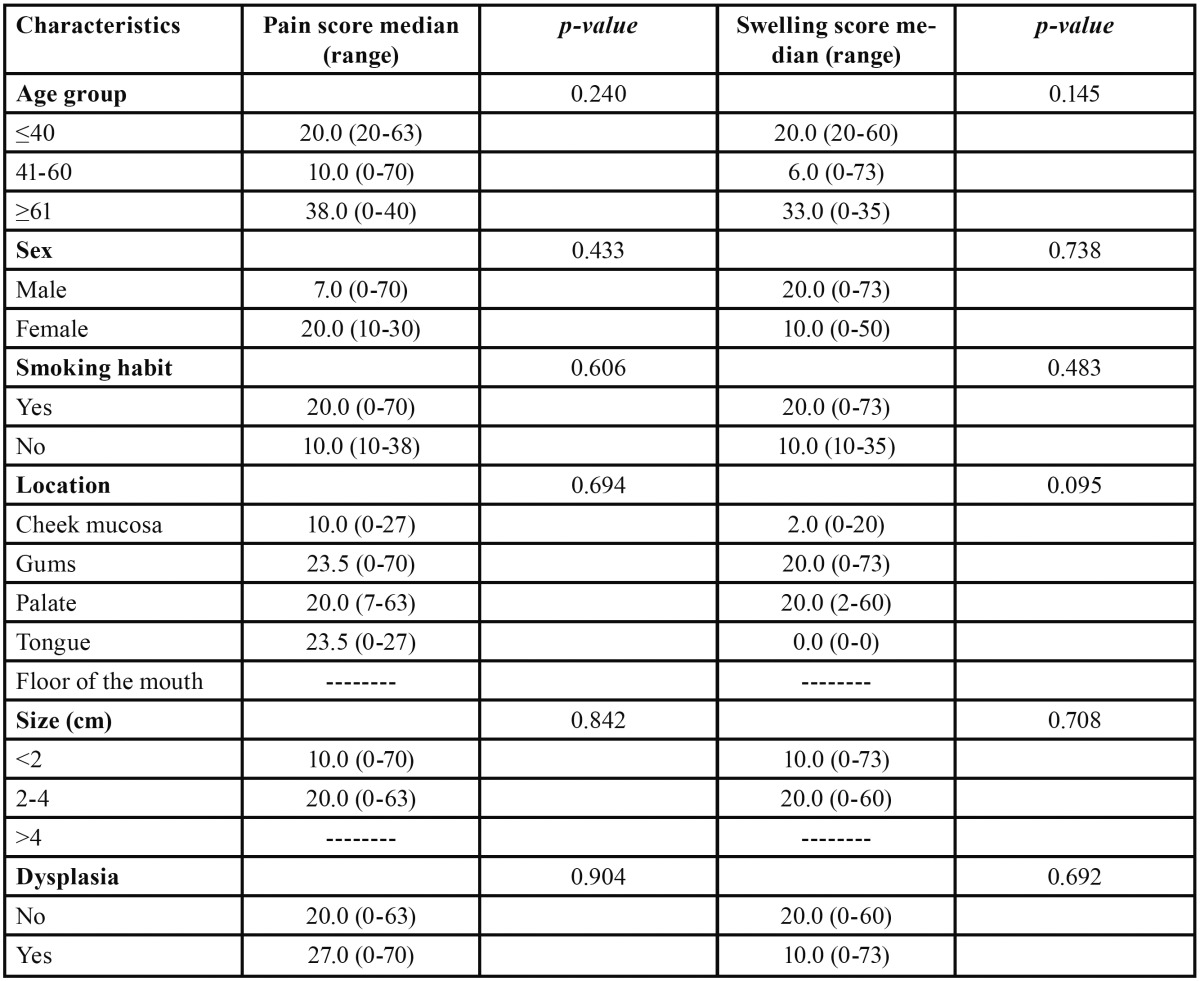
Comparison of median (range) pain scores (recorded at peak pain intensity 12 hours after surgery) and swelling scores (recorded at peak swelling intensity 24 hours after surgery) in the cold scalpel group (Kruskal-Wallis and Mann-Whitney U-test).

## Discussion

The present study involves two groups of patients with oral leukoplakia: one subjected to conventional surgical treatment with a cold knife and the other to CO2 laser surgery. In both groups the postoperative pain and swelling were evaluated.

The CO2 laser is currently accepted as the technique of choice in the management of many soft tissue lesions in the oral and maxillofacial regions. Compared to the scalpel, the CO2 laser affords a hemostatic effect particularly in highly vascularized areas. This in turn results in a clearer surgical field and therefore a reduction in operating time. Treatment with the laser has certain advantages, such as the selective removal of affected epithelium and minimal damage to surrounding healthy tissue, resulting in excellent wound healing with minimal or no scar tissue, and a good functional outcome (15-19). Other favorable features of CO2 laser therapy may include reduced postoperative pain and swelling.

In our study, the patients with oral leukoplakias treated with CO2 laser showed a lower postoperative pain and swelling than the patients treated with a cold knife, with statistically significant differences during the first three days after surgery. None of the patients showed malignant transformation during the period of follow-up (27.90 ± 12.05). Nevertheless, Schoelch et al. (20), after surgery treatment of 70 oral leukoplakias (48 homogeneous leukoplakia, 8 erythroleukoplakia and 14 verrucous leukoplakias) by mean CO2 laser, with a mean of period of follow-up of 32 months (range 6-178 months) saw that 5 patients (7.14%) developed a squamous cell carcinoma at the lesion site.

The most important disadvantage of CO2 laser evaporation of oral leukoplakia is the fact that the lesion is not available for histological study, in this sense an incisional presurgical biopsy must be obtained in all cases. The location of the lesion therefore must be carefully chosen. If the lesion is large or shows different clinical aspects, more than one biopsy is recommended (17-20).

One characteristic difference between the CO2 laser and cold knife is the generation of a coagulated tissue layer along the walls of the laser incision. This zone of thermal damage may impede the graft consolidation and reduce tensile strength during the period of wound healing, especially in extensive zones (17).

Measurement of postoperative pain is inherently difficult, as it comprises both physical and psychological aspects. Subjectively, the true character of pain experience is not directly accessible to the examiner. Therefore, the examiner must rely on the patient’s ability to communicate his or her perception and interpretation of the pain. In the laser group, on comparing the pain scores (recorded at peak pain intensity: between 12-24 hours after surgery) and swelling scores (recorded at peak swelling intensity: between 12-48 hours after surgery), no statistically significant differences were observed in relation to age, gender, smoking habit, location of lesion or dysplasia presence. Nevertheless, statistically significant differences were observed with respect to lesion size, with more pain and swelling in the most extensive lesions. In this sense, Van de Hem et al. (22), after surgical treatment of 282 oral leukoplakias (in 200 patients) with CO2 laser saw that the patients with most extensive lesions (≥2 cm) showed higher postoperative pain, and they recommended the vaporization in several sessions in patients with extensive lesions.

The main problem of oral leukoplakia is its possible malignant transformation, reported to occur in 1.2-9% of cases (6,19,24). Holmstrup et al. (18) found no evidence that surgical treatment is protective against cancer development and reported that surgical intervention does not appear to prevent oral premalignant lesions from developing into malignancy.

In an observational retrospective study published by Schepman et al. (23) compared the incidence of squamous cell carcinomas in two groups of subjects with oral leukoplakia: one comprising patients who received some type of active treatment (medical and/or surgical) and the other involving patients kept under regular clinical follow-up. No significant difference was observed in the risk of malignant transformation between the two groups. These results, perhaps suggesting that the natural history of oral leukoplakias might be independent of the treatment received and that there is a subgroup of lesions destined to undergo malignant transformation regardless of the therapeutic strategy adopted. Nevertheless, we always must realise a detailed study of the lesions and the higher risk lesions must be actively treated.

To date, our experience with the surgical removal of oral leukoplakia has been satisfactory with both of the described techniques in this study. Nevertheless, the studied sample is small and the follow-up period brief. These limitations must be taken into account and studies involving larger patient series and regular follow-up over longer periods of time are needed in order to derive more reliable information on the role of invasive surgical resection in the prevention of oral cancer in patients with oral leukoplakia.

In the present study, we found the CO2 laser to cause only minimal pain and swelling, thus suggesting that it may be an alternative method to conventional surgery in treating patients with oral leukoplakia.
